# Voluntary Physical Exercise Improves Subsequent Motor and Cognitive Impairments in a Rat Model of Parkinson’s Disease

**DOI:** 10.3390/ijms19020508

**Published:** 2018-02-08

**Authors:** Shih-Chang Hsueh, Kai-Yun Chen, Jing-Huei Lai, Chung-Che Wu, Yu-Wen Yu, Yu Luo, Tsung-Hsun Hsieh, Yung-Hsiao Chiang

**Affiliations:** 1 The Ph.D. Program for Neural Regenerative Medicine, College of Medical Science and Technology, Taipei Medical University, Taipei 11031, Taiwan; d620103001@tmu.edu.tw (S.-C.H.); chenkathryn@hotmail.com (K.-Y.C.); johnwu@tmu.edu.tw (C.-C.W.); yvonneyu524@hotmail.com (Y.-W.Y.); hsiehth@mail.cgu.edu.tw (T.-H.H.); 2 Center for Neurotrauma and Neuroregeneration, Taipei Medical University, Taipei 11031, Taiwan; m105095006@tmu.edu.tw; 3 Department of Surgery, School of Medicine, College of Medicine, Taipei Medical University, Taipei 11031, Taiwan; 4 Department of Neurosurgery, Taipei Medical University Hospital, Taipei 11031, Taiwan; 5 Department of Neurosurgery, Case Western Reserve University School of Medicine, Cleveland, OH 44106, USA; yxl710@case.edu; 6 Department of Physical Therapy and Graduate Institute of Rehabilitation Science, College of Medicine, Chang Gung University, Taoyuan 33302, Taiwan

**Keywords:** voluntary physical exercise, Parkinson’s disease, 6-hydroxydopamine, neuroprotection

## Abstract

Background: Parkinson’s disease (PD) is typically characterized by impairment of motor function. Gait disturbances similar to those observed in patients with PD can be observed in animals after injection of neurotoxin 6-hydroxydopamine (6-OHDA) to induce unilateral nigrostriatal dopamine depletion. Exercise has been shown to be a promising non-pharmacological approach to reduce the risk of neurodegenerative disease. Methods: In this study, we investigated the long-term effects of voluntary running wheel exercise on gait phenotypes, depression, cognitive, rotational behaviors as well as histology in a 6-OHDA-lesioned rat model of PD. Results: We observed that, when compared with the non-exercise controls, five-week voluntary exercise alleviated and postponed the 6-OHDA-induced gait deficits, including a significantly improved walking speed, step/stride length, base of support and print length. In addition, we found that the non-motor functions, such as novel object recognition and forced swim test, were also ameliorated by voluntary exercise. However, the rotational behavior of the exercise group did not show significant differences when compared with the non-exercise group. Conclusions: We first analyzed the detailed spatiotemporal changes of gait pattern to investigate the potential benefits after long-term exercise in the rat model of PD, which could be useful for future objective assessment of locomotor function in PD or other neurological animal models. Furthermore, these results suggest that short-term voluntary exercise is sufficient to alleviate cognition deficits and depressive behavior in 6-OHDA lesioned rats and long-term treatment reduces the progression of motor symptoms and elevates tyrosine hydroxylase (TH), Brain-derived neurotrophic factor (BDNF), bone marrow tyrosine kinase in chromosome X (BMX) protein expression level without affecting dopaminergic (DA) neuron loss in this PD rat model.

## 1. Introduction

Parkinson’s disease (PD) is the second most common neurodegenerative disorder after Alzheimer’s disease. It is a progressive neurodegenerative disorder in the central nervous system (CNS), characterized in part by dopaminergic (DA) neuron loss in the nigrostriatal system with clinical symptoms such as resting tremor, rigidity, akinesia, and disturbances of postural reflexes, affecting some 10 million people worldwide [1].

One of the hallmark changes of PD is shuffling gait and abnormal spatial indices of gait patterns. These gait abnormalities become pronounced in the advanced stages of PD, inducing further disability or limitation of mobility [2,3,4,5]. 6-OHDA unilateral lesion in rodent has been shown to recap these deficits. Recently, there are several techniques that have been developed to facilitate the analysis of the detailed gait parameters of PD rodent model [6,7,8], such as step length, stride length, base of support, foot angle, footprint area, and step sequence and cycle. Our previous study showed that the step length and stride length of 6-OHDA post-lesion animals become significantly shorter, especially on the contralateral side, whereas it is relatively consistent in the pre-lesion animals. Furthermore, we also observed that post-lesion rats had a wider base of support (BOS) than the pre-lesion rats by the ventral view of their footprints [6].

Physical exercise is an economical, practical, and relatively safe approach to developing neuroprotective and neurorestorative treatments for PD, because it can promote mitochondrial energy metabolism, upregulate antioxidant mechanisms, reduce inflammation, trigger angiogenesis, and produce synaptogenesis [9]. Studies have demonstrated protective effects of physical exercise against the behavioral and neuropathological effects of DA toxins [10,11]. In this study, we utilize the recently developed gait analysis technique to carry out a detailed characterization of gait abnormality in PD animals as well as to examine the effects of voluntary exercise on the gait parameters in PD animals.

There is increasing clinical evidence showing that the depression and cognitive deficits may affect up to 50% of patients with PD [12,13,14,15]. Dopamine replacement therapy such as Levodopa is the most effective treatment for PD targeting mainly motor symptoms, with less beneficial effects in non-motor symptoms [16,17]. Therefore, new therapeutic strategies to improve non-motor symptoms in PD are also urgently needed. Exercise has been thought to benefit cognitive and depressive functions of those with PD in both human and rodent [18,19,20,21,22], but the mechanism and which type of exercise impacts non-motor manifestation is still inconclusive. Therefore, in this study, we also examined the effects of voluntary exercise on non-motor functions such as cognitive function and depressive behavior in 6-OHDA lesioned rats.

## 2. Results

**Figure 1:**
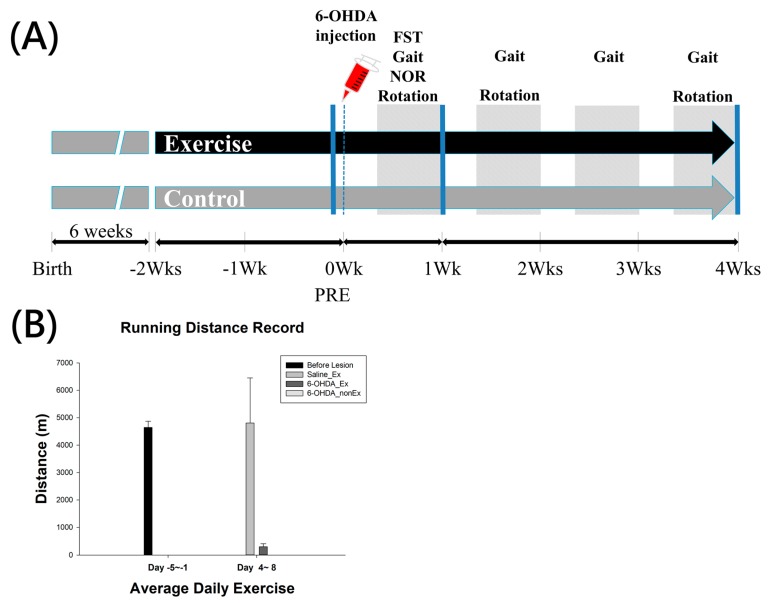
(**A**) Timeline of the experimental design; (**B**) The average running distance of each group before and after 6-OHDA lesion. 6-OHDA, 6-hydroxydopamine; NOR, Novel Object Recognition; FST, Forced Swimming Test; Wks, weeks.

The experimental design is shown in Figure 1. After one-week habituation, animals were separated into 6 groups: 6-OHDA + STE, 6-OHDA + LTE, 6-OHDA + non-Ex (STE control), 6-OHDA + non-Ex (LTE control), Saline + STE, and Saline + non-Ex (STE control). The number of animals in each group is described in Materials and Methods. Following two weeks’ exercise, rats were given 6-OHDA or saline, and continued running for one week (STE) or four weeks (LTE) to evaluate the short-term neuroprotection and long-term neurorestorative effects of exercise. After behavioral evaluations, all rats were euthanized for immunohistochemical and immunoblotting investigations.

### 2.1. Behavioral Amelioration in Parkinson’s Disease Model of Rats Receiving Exercise-Novel Object Recognition (NOR), Rotational Test (RT) and Forced Swimming Test (FST)

**Figure 2:**
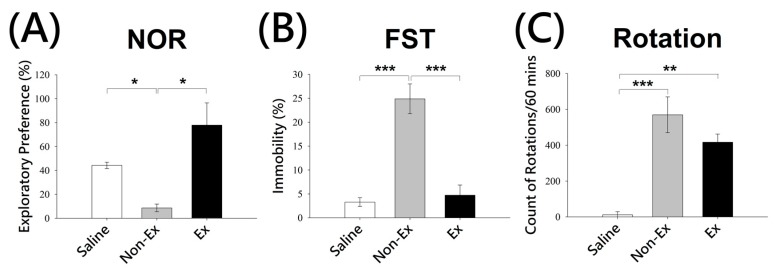
Effects of voluntary exercise on behavioral assessments in animals exposed to 6-OHDA. Animals were subjected to novel object recognition (NOR) test (**A**); forced swimming test (FST) (**B**); and rotational test (**C**). Values were expressed as mean ± SEM (NOR, *n* = 4; FST, *n* = 4; Rotation, *n* = 5 (1 week, Wk)) compared with the saline or non-exercise (Non-Ex) group. All exercises and non-Ex groups were injected with 6-OHDA and saline group is injected with saline as control. All analyses based on one way ANOVA, * *p* < 0.05; ** *p* < 0.01; *** *p* < 0.001.

During NOR, significant differences were observed between the exercise and non-Ex groups in 6-OHDA lesioned rats. Unilateral 6-OHDA lesion in rats resulted in significant deficits in recognition of a novel object as shown by the significant difference observed in non-lesioned and lesioned rats that did not undergo exercise (Discrimination Index: Saline + STE, 44.27 ± 2.7%; lesioned Non-Ex, 8.6 ± 3.2%, *p* < 0.05, ANOVA, Figure 2A). Importantly, the lesioned-exercise group showed increased exploratory preference towards novel object compared to the non-Ex group (lesioned Exercise, 77.85 ± 18.58%, *p* < 0.05, ANOVA, Figure 2A), suggesting exercise is able to ameliorate the cognitive deficits caused by 6-OHDA lesion. In the FST, the immobility time demonstrated significant differences between the depression-like behavioral patterns of the animals after exercise. Statistical analyses showed an increase in the percentage of total time animals were immobile in the lesioned non-Ex group (24.88 ± 3.11%) compared with the saline group (3.3 ± 0.9%, *p* < 0.001, ANOVA, Figure 2B). However, lesioned rats subjected to exercise showed reduced immobility in FST compared to non-Ex group (4.73 ± 2.12% vs. 24.88 ± 3.11%, *p* < 0.001, ANOVA, Figure 2B), suggesting exercise is also able to reduce the depression-like behaviors caused by 6-OHDA lesion in rats. In the RT, a significant increase in the number of rotations was observed for the exercise and non-Ex groups compared with the saline group, although there was no significant difference between the exercise and non-Ex group (Saline, 12.2 ± 16.7 turns/h; Non-Ex, 569.6 ± 99.7 turns/h; Exercise, 416.6 ± 45.9 turns/h, *p* = 0.152, ANOVA, Figure 2C).

### 2.2. Spatiotemporal Gait Analysis

Seventeen lesioned-exercise rats and twenty-one lesioned non-Ex rats completed gait analysis before lesion (on the second week of exercise) and within one-week and four-week post-lesion.

**Figure 3:**
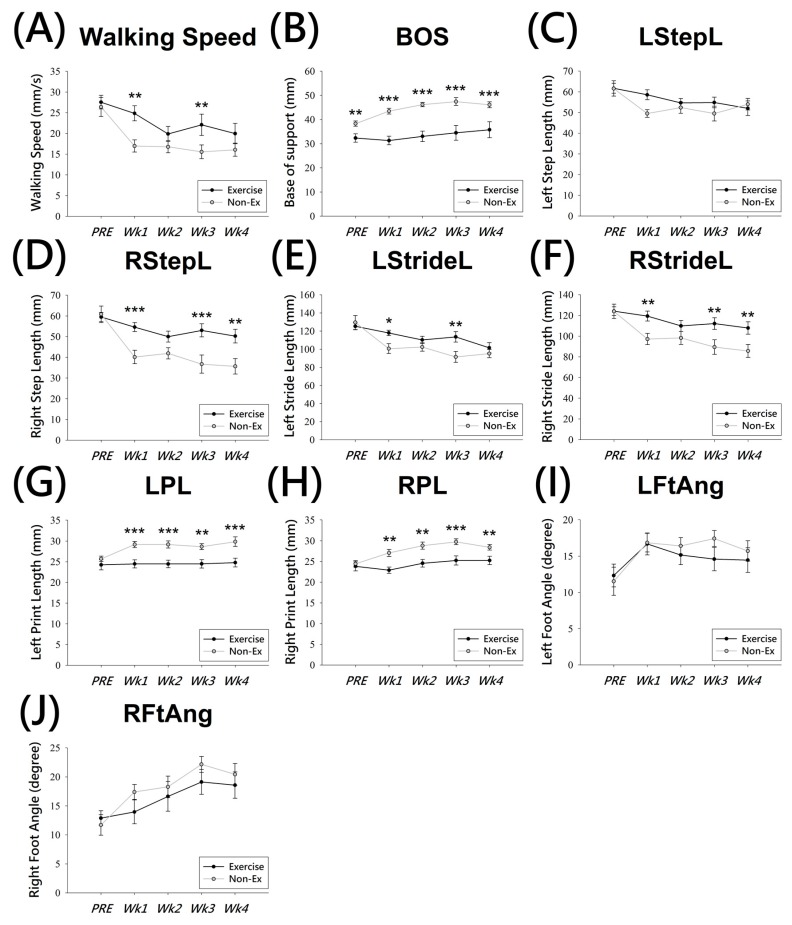
Effects of voluntary exercise on gait analysis in animals exposed to 6-OHDA. Using twelve parameters to determine the gait disturbance changes in animals after administration of 6-OHDA, which induced unilateral nigrostriatal dopamine depletion. Walking speed (**A**); base of support (BOS) (**B**); left step length (LStepL) (**C**); right step length (RStepL) (**D**); left stride length (LStrideL) (**E**); right stride length (RStrideL) (**F**); left print length (LPL) (**G**); right print length (RPL) (**H**); left foot angle (LFtAng) (**I**); right foot angle (RFtAng) (**J**). Values were expressed as mean ± SEM (Non-Ex, *n* = 15; Exercise, *n* = 12) and compared with the pre 6-OHDA lesion (PRE) or non-exercise (Non-Ex) group. All analyses based on two way ANOVA, * *p* < 0.05; ** *p* < 0.01; *** *p* < 0.001.

For walking speed, the post hoc multiple comparison test showed that the walking speed in post-lesion animals was markedly reduced (*p* < 0.001) (Figure 3A), and the two way ANOVA showed a significant difference between the exercise and non-Ex groups at 1 week and 3 weeks post-lesion (*p* < 0.01) (Figure 3A). The average walking speed was 26.39 ± 2.29 cm/s (non-Ex) and 27.54 ± 1.64 cm/s (exercise) in the pre-lesion state, which was markedly decreased at the post-1 week. The speed was approximately 16.97 ± 1.48 cm/s (non-Ex) vs. 24.85 ± 1.82 cm/s (exercise) (*p* < 0.001). The BOS of the Ex-group, 33.386 ± 0.86 mm, was statistically lower than the non-Ex-group, 44.352 ± 0.77 mm (*p* < 0.001) (Figure 3B). The post-lesion rats in the non-Ex group showed a significantly larger BOS compared to pre-lesion levels (*p* < 0.001) (Figure 3B), and also larger than Ex group at all time points. Interestingly, pre-lesion (PRE) levels of BOS showed significant differences, 32.34 ± 1.75 mm (Ex) and 38.3 ± 1.08 mm (non-Ex), (*p* < 0.001) between the two groups.

With regard to the step and stride lengths, these two parameters were decreased bilaterally in the post-lesion animals (left step length, LstepL, 56.342 ± 1.34 mm (Ex), 53.569 ± 1.2 mm (non-Ex), *p* = 0.124; right step length, RstepL, 53.456 ± 1.56 mm (Ex), 42.35 ± 1.39 mm (non-Ex), *p* < 0.001; left stride length, LstrideL, 113.69 ± 2.5 mm (Ex), 104.228 ± 2.23 mm (non-Ex), *p* < 0.01; right stride length, RstrideL, 144.625 ± 2.78 mm (Ex), 98.32 ± 2.48 mm (non-Ex), *p* < 0.001) (Figure 3C–F). Animals that showed DA neuron degeneration have an imbalanced gait pattern, and the step and stride lengths are shorter than control animals. However, the comparison between the two groups (Ex and non-Ex) also showed significant differences at 1, 3, and 4 weeks post-lesion (Figure 3D,F) on the contralateral (right) side of their body, and the ipsilateral (left) side was not so different (Figure 3C,E). It is worth noting that step length of the right side, RstepL (Figure 3D), decreased much more than that of the left side (Figure 3C). Furthermore, the difference in stride length of the right side, RstrideL, revealed a higher recovery trend (Figure 3F) compared with the left side, LstrideL (Figure 3E).

In addition, the print length (PL), left PL (LPL) and right PL (RPL) (Figure 3G,H), showed a significant difference between Ex (LPL, 24.489 ± 0.43 mm; RPL, 24.353 ± 0.41 mm) and non-Ex (LPL, 28.224 ± 0.39 mm; RPL, 27.656 ± 0.36 mm) groups at the post-lesion time points (*p* < 0.001). However, the foot angle, LFtAng, and RFtAng (Figure 3I,J), in the Ex and non-Ex groups showed no significant differences at each post-lesion time points.

### 2.3. TH Immunohistochemistry

**Figure 4:**
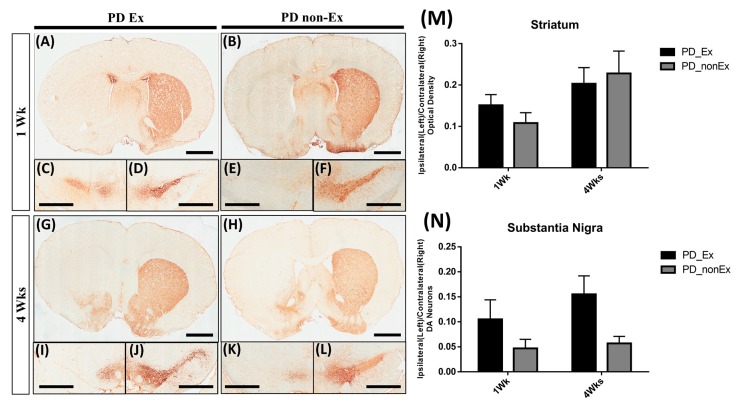
Tyrosin Hydroxylase (TH) immunostaining in the striatum and substantia nigra at 1 week (Wk) and 4 weeks (Wks) post lesion. There is no significant differences in TH immunoreactivity in the striatum (**A**,**G**) and substantia nigra (**C**,**D**,**I**,**J**) of rats in the exercise group, compared to that of rats in the non-exercise group ((**B**,**H**): striatum; (**E**,**F**,**K**,**L**): substantia nigra). The optical density of striatum (**M**) and DA neurons of SN (**N**) are quantified and shown on the right. Scale bar, 2 mm (**A**,**B**,**G**,**H**); 1 mm (**C**–**F**,**I**–**L**).

We investigated the expression of TH in the striatum under the following conditions: post-lesion 1 week (1 Wk) (Figure 4A,B), post-lesion 4 Wk (Figure 4G,H), and in the SN, post-lesion 1 Wk (Figure 4C–F), post-lesion 4 Wk (Figure 4I–L). The 6-OHDA-injected lesioned left brain showed significantly decreased TH positive immunoreactivity in both striatum and SN, when compared with the intact striatum and SN of the right side. TH positive immunoreactivity in the lesioned striatum was not significantly increased by exercise (Figure 4A,G), as compared with the PD non-exercise group (Figure 4B,H). The positive immunoreactivity of the striatum and DA neurons of SN were shown in Figure 4M,N. Although there was no significant difference between Ex and non-Ex groups, we still observed some TH positive immunoreactive structures were preserved by exercise at 1-week post-lesion (Figure 4C).

### 2.4. Upregulation of BMX (Bone Marrow Tyrosine Kinase in Chromosome X), Brain-Derived Neurotrophic Factor (BDNF), and TH (Tyrosine Hydroxylase) Expression in the Striatum of Rats Receiving Exercise

**Figure 5:**
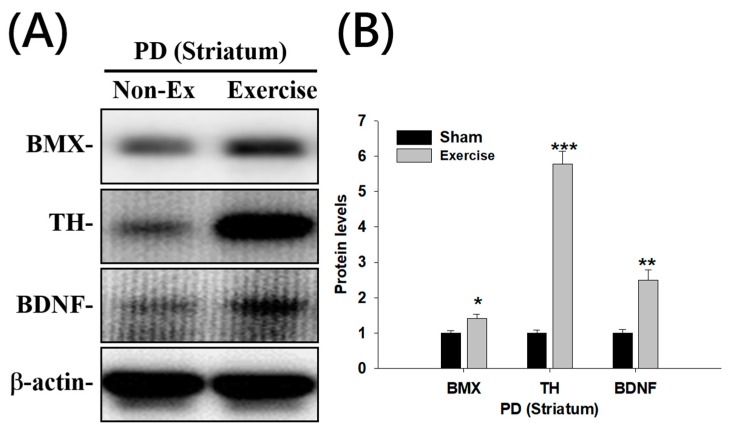
Effects of physical training on bone marrow tyrosine kinase in chromosome X (BMX), tyrosine hydroxylase (TH), and Brain-derived neurotrophic factor (BDNF) expression levels at 1 week (Wk) after lesion. Immunoblotting was performed to detect the changes in the expression of BMX, TH, and BDNF in the ipsilateral side of striatum of the animals exposed to 6-OHDA (**A**). Values were expressed as mean ± SEM (*n* = 6), * *p* < 0.05, ** *p* < 0.01, and *** *p* < 0.001, and then compared with Non-Ex group according to two-way ANOVA followed by Tukey’s post-hoc test (**B**).

To investigate for potential factors that might contribute to the beneficial effects caused by voluntary exercise, we examined the expression levels of several candidate proteins in the lesioned striatum in non-Ex or exercise groups by western blot. Our results show that following exercise, epithelial and endothelial tyrosine kinase/bone marrow tyrosine kinase in chromosome X protein (Etk/Bmx), BDNF, TH (Figure 5), were all upregulated on the lesioned side of the striatum (BMX: 1.41 ± 0.12, *p* < 0.05; TH: 5.79 ± 0.35, *p* < 0.001; BDNF: 2.50 ± 0.29, *p* < 0.01), compared to those of non-Ex group (BMX: 1.00 ± 0.07; TH: 1.00 ± 0.08; BDNF: 1.00 ± 0.11) (Figure 5).

## 3. Discussion

The present study investigated the changes in depression-like, cognitive, rotational, and gait behavior by exercise, including FST, NOR, rotation, gait spatiotemporal patterns following unilateral 6-OHDA brain lesions in rats.

Although PD and depression have distinct etiologies, PD is associated with depression [23]. Some studies have shown that cognitive impairments, such as recognition memory loss, can occur before the motor symptoms [24,25]. Furthermore, several experimental studies have demonstrated behavioral changes in animal models of PD, including depression [26,27] and reduced cognitive function [20]. In our study, we observed that voluntary running wheel exercise treatment can effectively alleviate the impairment of depressive-like and cognitive behaviors in our rat PD model.

Several animal models of PD have been established, including the neurotoxin 6-OHDA model [28]; however, the exact mechanisms for 6-OHDA-induced depressive behavioral and cognitive deficits are still not well known. It also has been shown that PD patients have learning and memory impairments, and the nigrostriatal [29] and mesocortical [30] pathways are involved in these cognitive deficits. The NOR test has been widely used to study recognition memory deficits in the PD rodent models. In our study, we observed that voluntary running wheel exercise could significantly ameliorate the depressive-like and cognitive impairment caused by 6-OHDA (Figure 2). Although we did not see a significant difference in the apomorphine-induced rotation test, there was a tendency toward a subtle decrease in rotations in the exercise group, suggesting that exercise could have a modest neuroprotective effect against this neurotoxin insult. The reason that we did not observe the difference between these two groups may be because the dose of apomorphine was too high to show supersensitivity differences, and 0.1 mg/kg body weight would be more appropriate, rather than 0.5 mg/kg [28]. The mechanism of the effect of voluntary exercise may involve increasing the levels of BMX and BDNF in the striatum, resulting in a decrease in 6-OHDA-induced toxicity. BDNF is a well-known molecule that regulates neuronal survival, differentiation, and synaptic plasticity, and many studies have shown its upregulation through exercise [10,26,31,32].

The epithelial and endothelial tyrosine kinase/bone marrow tyrosine kinase gene in chromosome X protein (BMX), a member of the Tec (tyrosine kinase expressed in hepatocellular carcinoma) family of non-receptor tyrosine kinases, is present in epithelial, endothelial, and granulomonocytic cells [33]. BMX and its family members have been shown to be major modulators of signaling pathways initiated by various extracellular stimuli, including growth factors, cytokines, hormones, and extracellular matrix molecules [34]. Several reports have validated the importance of BMX in differentiation, proliferation, cell motility, and apoptosis [35,36]. Current studies of Bmx primarily focus on their roles in cancer, whereas their endogenous function is still less studied. In our previous studies, we found that BMX induces activation by H2O2 in primary cortical neurons and plays an important role in ischemia-induced cell death [37]. In this study, we found that BMX expression was induced by exercise in the striatum.

Gait disorders are commonly observed in patients with PD resulting from the degeneration of DA neurons in the SN [38]. In this study, we used the video-based gait analysis system, which has been published before [6,8], and the major difference to the CatWalk XT system was the image resolution. We analyzed only the hindlimb of each rat; hence, we did not obtain their step sequence parameter. A study showed that the rat step sequence preference changes after a unilateral 6-OHDA lesion [7]. In our study, we evaluated both sides of the gait pattern and found that not only the lesioned side, the right side, but also the non-lesioned side, the left side, were both impaired in the parameters of step length, stride length, and print length (Figure 3C–H). This suggests that the bilateral coordination of muscle movement is involved in gait behavior. The measurements of gait patterns and behavioral tests indicate that exercise can reduce these gait deficits in 6-OHDA-treated animals. Our results showed that dopamine deficiency induced significant changes in walking speed, BOS, step/stride length, print length, and foot angle after 1–4 weeks post-lesion. After the unilateral injection of 6-OHDA, a bilateral impairment in gait performance appeared at 1-week post-lesion. However, we observed that the affected side showed a more severe deficit than the unaffected side (Figure 3). It suggests that the gait disturbances on one side would interact with the other side. Importantly, our data showed that physical exercise can significantly ameliorate gait impairments induced by 6-OHDA on both sides.

## 4. Materials and Methods

### 4.1. Animals

All rats were obtained from the Laboratory Animal Center (LAC) of National Defense Medical Center (NDMC), Taiwan. The rats were housed at 25 °C in a 12 h/12 h light/dark cycle with continuous water and food supply. All efforts were made to reduce animal suffering and minimize the number of animals used. This study was approved by the Institutional Animal Care and Use Committee (IACUC). Protocol number, LAC 100-0219, approve on 20 June 2012.

Animal studies were conducted on 71 adult female Sprague Dawley rats with a range of body weights between 200 and 300 g and ages between 8 and 12 weeks at experimental onset. The animals were separated into two groups, exercise (Ex) and non-exercise (non-Ex). A total of 38 rats were assigned for the short-term exercise (STE) experiment, with 21 in the non-Ex control group and 17 in the Ex group with 3 weeks of exercise (2 weeks of pre-exercise and 1 week of exercise after 6-OHDA lesion). Additionally, 27 rats were used for the long-term exercise (LTE) experiment, with 15 in the non-Ex control group and 12 in the Ex group with 6 weeks of exercise (2 weeks of pre-exercise and 4 weeks of exercise after 6-OHDA lesion). There were 6 rats in a saline control group; 3 were in the non-Ex group, and 3 were in the Ex group. All rats in the saline group were only used for the STE experiment.

### 4.2. Exercise

All Ex group animals were caged in transparent Plexiglas arenas (43 × 43 × 43 cm^3^), and each cage contained one running wheel (35 × 17 × 37 cm^3^; diameter, 32 cm) and one rat only. Runners had unlimited access to a running wheel in their cage for 3 or 6 weeks. We used a bicycle speed meter to record the daily rotations and then converted the numbers of rotations to kilometers.

### 4.3. Chronic Hemi-Parkinsonian Rat Model

For the 6-OHDA lesion, rats were anesthetized with intraperitoneal 50 mg/kg Zoletil 50 and 2 mg/kg Rompun, and placed into a stereotactic apparatus (Stoelting, Wood Dale, IL, USA) by using 45° non-puncture ear bars. In order to destroy nigrostriatal pathway, 2 mg/mL of 6-OHDA in 0.02% ascorbic saline (Sigma Chemical Co., St. Louis, MO, USA) was injected into the media forebrain bundle (MFB) on the left side of the brain using a 26-gauge 10-μL Hamilton microsyringe [6]. Total 4 μL of 6-OHDA was injected with a syringe pump through a rate of 0.5 μL/min. To avoid the 6-OHDA backfilling along the needle track, the needle was left in the brain for 5 min after injection. After the 6-OHDA lesion, we used the apomorphine-induced rotational test to verify the effectiveness at 1 to 4 weeks post-lesion. The apomorphine solution (Sigma-Aldrich: 0.5 mg apomorphine in 0.1% ascorbate saline), 0.5 mg/kg, was injected subcutaneously and we recorded the rotation for 60 min after injection. We excluded the rats that did not show apomorphine-induced contralateral rotation behaviors.

### 4.4. Novel Object Recognition (NOR)

Before the acquisition phases, rats were habituated in the open field box and then transferred to their home cage. Next, rats were placed back into the box after the addition of two objects of the same material placed in a symmetrical position, for 10 min. After 1 h, one of the objects was replaced with a novel object. Exploration was defined as rearing on the object, sniffing it at a distance of less than 2 cm, and/or touching it with the nose. Data were analyzed and expressed as the time spent at each object and as an Aggelton Discrimination Index (ADI; Aggleton et al., 1997) [39], defined as: (Time spent at the novel object − Time spent at the familiar object)/(Time spent at the novel object + Time spent at the familiar object).

### 4.5. Spatiotemporal Analysis of Gait Patterns

A walking track equipped with a video-based system was modified for obtaining spatiotemporal parameters of gait in this study. The walking track device consisted of a Plexiglas chamber 80 (l) × 6 (w) × 12 (h) cm^3^ with a mirror sloped at 45° underneath the walking track, allowing for observations with recording by a high-speed digital camera (EX-F1, Casio, Iruma City, Japan).

Before the experiment, rats were familiarized with the walking track by allowing them to walk freely on the track for 20 min before the test. The walking task was repeated 5 or 6 times in each direction, and complete walks of at least 4 steps were recorded without pause. The digital images of hindlimb stepping patterns captured from each walking trial were conducted with a threshold setting using MATLAB software (MathWorks, version 7.6, R2008a, Natick, MA, USA). After the sequential footprints were identified, we determined four spatial parameters: the step length, the stride length, the base of support (BOS), and the foot angle; and three temporal gait parameters: the walking speed, the stance/swing phase time, and the stance/swing ratio. Each parameter was averaged for at least 20 footsteps [6].

### 4.6. Forced Swimming Test (FST)

The experiment consisted of two sessions in a transparent cylindrical tank of 35 cm in diameter and 70 cm in height: an initial 5-min test followed by a second 5-min test for quantitation (see below) performed 24 h later. During the test, the rat’s behavior was observed by video recording, and the time of immobility and swimming was measured. A rat was regarded as immobile when floating motionless or making only small adjustments necessary to keep its head above the water. A rat regarded as swimming was defined by horizontal movements of an animal around the tank.

### 4.7. Fixation and Sectioning

The animals perfused transcardially with 0.9% saline and 4% paraformaldehyde (PFA) in 1× phosphate buffer (PBS). After PFA perfusion, brains were removed and post-fixed for 1 day and dehydrated in 20%, and then 30% sucrose until the brain sank. The brains were cut into 10-μm sections containing the Str and the SN on a cryostat. Every 8 sections were selected from a region spanning from −5.20 mm to −5.80 mm in the SN and from +1.70 to +2.30 mm in the Str with respect to the bregma.

### 4.8. Immunohistochemistry and Immunofluorescence

The frozen sections were washed in PBS and then incubated in 0.2% Triton X-100 in PBS for 15 min. After PBS washing, the sections were blocked with 3% normal goat serum and 3% BSA for 1 h. The sections were subsequently incubated with a 1:200 dilution of rabbit primary anti-TH (cat #AB125, Millipore, Burlington, MA, USA) in blocking buffer overnight at 4 °C. After the primary antibody incubation, the biotinylated secondary antibody and streptavidin conjugated horseradish peroxidase (HRP) were used to amplify the signal. We then used substrate chromogen diaminobenzidine (DAB) (Dako, Glostrup, Denmark) to visualize the HRP expression. The striatal TH-positive fibers optical density was measured by using Image-Pro Plus software in each section, and the TH-positive neurons in the SN were manually counted in each section on both hemispheres. Both striatal TH-positive fibers optical density and the SN TH-positive neurons in the ipsilateral side were calculated and normalized with the contralateral side.

### 4.9. Immunoblotting

Striatal tissue was first lysed by adding RIPA buffer with protease inhibitor cocktails; 50 μg proteins in tissue lysates were diluted in 5× SDS buffer and denatured at 95 °C for 5 min. Proteins were then electrophoresed on 10 or 12% SDS-polyacrylamide gel and electro-transferred onto a polyvinyldifluoride membrane. After 1 h incubation with blocking solution, the membranes were incubated with primary antibodies overnight. After washing three times, the membranes were incubated with goat HRP-linked anti-mouse and anti-rabbit IgG antibody for 1 h and then developed with an enhanced chemiluminescence plus detection kit (Amersham Life Sciences, Piscataway, NJ, USA). All results were normalized to the levels of beta-actin used as the loading control, and the amount of immunoreactivity calculated relative to the expression with the corresponding controls.

### 4.10. Statistical Analysis

The two-way repeated measure analysis of variance (ANOVA) was used to test both group and time factors in gait analysis and immunohistochemistry quantification. If the main effect of time was significant, multiple within-subject comparisons will be taken with the Bonferroni correction post hoc test. For NOR, FST and rotation, a one-factor analysis (group) repeated-measures ANOVA was used to compare saline, Ex and non-Ex group at 1 week post-lesion point followed by a Bonferroni correction post hoc test. For immunoblotting, the pair t-test was done to measure the differences between the Ex and non-Ex group. Data were analyzed using Sigma Plot version 12.5 (Systat Software Inc., San Jose, CA, USA) with the significance level set at *p* < 0.05 for each calculation. All data are shown as the average ± standard error of the mean (SEM).

## 5. Conclusions

We have found that exercise has protective effects on the depression-like behavior, cognition, and gait impairments induced by the neurotoxin 6-OHDA at early time points. We further analyzed the gait phenotype in a PD rat model at a long-term time point, 4 weeks after 6-OHDA lesion, and observed a significant improvement in locomotor behavior. Protein expression analysis further documents the importance of BMX and BDNF levels induced by exercise, which suggests potential pathways that might contribute to the behavioral improvement. Future research may utilize the behavioral methodology described here to better understand the mechanism of exercise-induced neuroprotection, and the development of novel treatment protocols to improve functional recovery in both motor and non-motor function in PD.

## Abbreviations

PD	Parkinson’s Disease
6-OHDA	6-Hydroxydopamine
TH	Tyrosine Hydroxylase
DA	Dopaminergic
SN	Substantia Nigra
